# Antioxidant defense system responses, lysosomal membrane stability and DNA damage in earthworms (*Eisenia fetida*) exposed to perfluorooctanoic acid: an integrated biomarker approach to evaluating toxicity

**DOI:** 10.1039/d1ra04097a

**Published:** 2021-08-03

**Authors:** Zhifeng Wang, Chaona Li, Yuanyuan Shao, Weina Xue, Ning Wang, Xiaoming Xu, Zhibin Zhang

**Affiliations:** School of Municipal and Environmental Engineering, Shandong Jianzhu University No. 1000 Fengming Road Jinan 250101 P. R. China wangzhifeng18@sdjzu.edu.cn; Jiangxi Nuclear Industry Geological Bureau Testing Center No. 101 Hongduzhong Avenue Nanchang 330002 P. R. China

## Abstract

Perfluorooctanoic acid (PFOA) is one of the most representative perfluoroalkyl substances and has garnered intense human and ecological health concerns due to its ubiquity in the environment, bio-accumulative nature and potential toxicological effects. In this study, an artificial soil containing PFOA was used to evaluate the biological toxicity of PFOA to earthworms *Eisenia fetida*. Six kinds of oxidative stress biomarkers, including superoxide dismutase (SOD), catalase (CAT), glutathione peroxidase (GPx), glutathione S-transferase (GST), reduced glutathione (GSH) and lipid peroxidation (LPO), as well as lysosomal membrane stability (LMS) and DNA damage in earthworms were detected after exposure to 0, 10, 20, 40, 80 and 120 mg kg^−1^ PFOA in the soil for 3, 7, 14, 28, and 42 days. The results of multi-biomarker responses indicated that PFOA can induce various adverse effects on earthworms, including growth inhibition, oxidative stress and genotoxicity, resulting in lipid membrane peroxidation, decreased lysosomal membrane stability and DNA damage. LPO, LMS and DNA damage all presented dose- and time-dependent relationships. An integrated biomarker response (IBR) index was applied to summarize the multi-biomarker responses to star plots, and the IBR value was calculated as the area of the plots to indicate the integrated stress of PFOA on earthworms. The IBR index showed that the integrated stress induced by PFOA increased markedly throughout the exposure period, exhibiting a concentration-related and exposure time-related effect. The graphical changing trend of the IBR star plots, along with the multi-biomarker responses, suggested that the biomarkers of the antioxidant defense system in earthworms are sufficiently sensitive for short-term PFOA biomonitoring programs, while the bioindicators that indicate actual damage in organisms are more suitable to be employed in long-term monitoring programs for the risk assessment of PFOA. This is the first study evaluating the biological toxicity of PFOA by using an integrated biomarker approach. Our results showed that PFOA can potentially damage soil ecosystems, which provides valuable information for chemical risk assessment of PFOA in the soil environment and early warning bioindicators of soils contaminated by PFOA.

## Abstract 2

The integrated biomarker response (IBR) index was calculated to evaluate the integrated toxicological effects of PFOA on earthworm *Eisenia fetida*.

## Graphical abstract

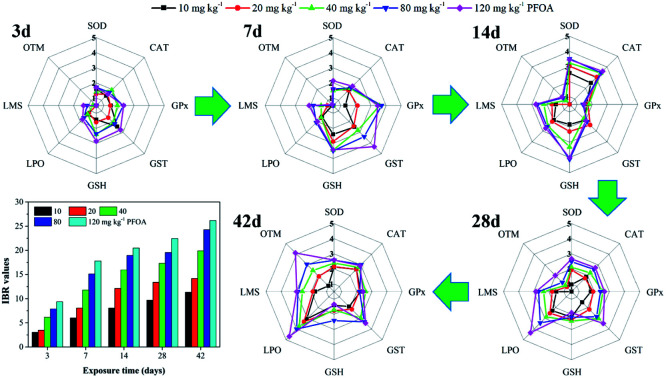

## 1. Introduction

Perfluorooctanoic acid (PFOA, CF_3_(CF_2_)_6_COOH), one of the most representative perfluoroalkyl substances, has been produced and used worldwide for over six decades in a variety of commercial and industrial products such as food packaging, cosmetics, fire-fighting foams, stain-resistant textiles and fabrics, and surface protecting agents for paper.^1–3^ Consequently, PFOA has been broadly detected in the environment, including indoor air, water, sediment, and soil, as well as in wildlife and human tissues.^4–9^ Due to its extraordinary persistence, bio-accumulative nature and potential toxicological effects, PFOA has garnered intense human and ecological health concerns.^10–12^ General toxicological findings associated with exposure to PFOA include potential neurotoxicity, molecular toxicity, hepatotoxicity, immunotoxicity, hormonal effects and carcinogenic potential.^13,14^ However, most of the published research on toxicological effects of PFOA so far has mainly focused on rodents,^15,16^ aquatic organisms,^17–19^ humans^20,21^ or other wildlife,^22,23^ while very few studies have been conducted on soil microfauna. In order to develop a comprehensive ecotoxicity profile for PFOA in soil environment, the toxicity of PFOA-contaminated soils should be assessed by using suitable model organisms.

Earthworms, which comprise the largest part of the soil fauna biomass, are sentinels for terrestrial systems. Their activities are essential for the mixture and translocation of soil constituents.^24^ Due to their close interaction with soil, earthworms can be profoundly affected by soil pollution and accumulate contaminants in the body. These features make them ideal model organisms for ecological risk assessment of toxic substances under controlled and natural conditions.^25,26^ Given that the species *Eisenia fetida* is available commercially, susceptible to chemicals and easily bred under laboratory conditions, it has been recommended for this purpose in the Organization for Economic Co-operation and Development (OECD) exposure protocol,^27^ and was chosen in this study for ecotoxicological assays.

The molecular, subcellular, cellular and physiological levels of earthworms change significantly when they are under contamination stress. Each type of these biological responses produces a specific biological signal, called a biomarker. In the past few years, there has been a noticeable increase in the use of biomarkers of earthworms to assess the impacts of contaminants on terrestrial ecosystem.^28,29^ The oxidative stress biomarkers, including antioxidant enzymes, non-enzymatic antioxidants and lipid peroxidation level are frequently applied to explore the toxicity mechanism of many perfluoroalkyl substances (PFASs). Yuan *et al.*^30^ evaluated the effects of perfluorooctane sulfonate (PFOS) and PFOA on superoxide dismutase (SOD) in the earthworm *E. fetida* at sublethal concentrations and found that SOD activity was sensitive to evaluate the toxicity of PFOS- and PFOA-contaminated soils. Zhao *et al.*^31^ also assessed the potential toxicity of PFOA to the earthworm *E. fetida* by measuring the responses of SOD, catalase (CAT), glutathione S-transferase (GST) and lipid peroxidation (LPO) after exposure to 0, 5, 10, 20, and 40 mg kg^−1^ PFOA in soils for 7, 14, 21, and 28 days. The results indicated that PFOA has adverse biochemical effects on *E. fetida*. The oxidative stress biomarkers in human liver cells (HepG2) have also been applied to investigate the toxic mechanism of many PFASs. Wielsøe *et al.*^32^ found that the oxidative stress increased significantly in HepG2 exposed to seven long-chained PFASs in an exposure time of 24 h. Ojo *et al.*^33^ investigated the combined effects of five PFASs on HepG2 for 24 h by using an orthogonal design. The results showed that both individual and combined PFASs could induce concentration-dependent cytotoxicity and depletion of reduced glutathione (GSH) levels. Apart from the above molecular biomarkers, lysosomal membrane stability (LMS) and DNA damage have been used as subcellular biomarkers of earthworm coelomocytes because they can detect the sensitive physiological response of earthworms to toxic pollutants. The neutral red retention time (NRRT) assay sensitively detects decreased lysosomal membrane stability,^34,35^ and the comet assay is sufficiently sensitive to indicate DNA damage by bioindicators of tail DNA%, tail DNA length (TL) and olive tail moment (OTM).^36,37^ Zheng *et al.*^37^ applied an artificial soil method to study the effects of PFOS and PFOA on earthworm *E. fetida*, and DNA damage was detected in the organism after 14 d acute exposure. Xu *et al.*^38^ also conducted an artificial soil test to investigate the potential toxicity of PFOS to earthworm *E. fetida* and found that PFOS could induce damage in earthworm coelomocytes; the OTM, tail DNA% and TL values increased significantly with the PFOS concentration in soils. However, up to now, few studies have focused on the potential toxicity of PFOA to earthworms by using biomarkers at different levels simultaneously.^37^

Previous studies have proved that no single biomarker can provide all the information necessary to evaluate exposure or its significance. Furthermore, the responses of one biomarker provide information that improves interpretation of other biomarkers.^39^ Therefore, the application of a battery of biomarkers is more effective in evaluating the effects of contaminant exposure and assessing environmental pollution stress when compared with the use of a single biomarker.^40,41^ Aarab *et al.*^41^ collected fish (chub, barbel and trout) in 11 sites in rivers in south-west France and measured five biomarkers in muscle or brain for acetylcholinesterase (AChE) and in liver for GST, CAT and 7-ethoxyresorufine-*O*-deethylase (EROD). The sites were clearly discriminated according to a multi-biomarker pollution index calculated as the sum of the response index. In another study conducted by Beliaeff and Burgeot,^42^ a battery of biomarkers were measured in mussel and fish to evaluate the effects of exposure to polycyclic aromatic hydrocarbons (PAHs) and polychlorobiphenyls (PCBs). The biomarker responses of AChE, GST, CAT, EROD and DNA adducts were computed as the star plot, and an integrated biomarker response (IBR) index was calculated as the plot area. The IBR method appeared to be a useful tool as an indicator of environmental pollution stress. In this study, the multi-biomarker approach, IBR index,^42^ was also calculated to summarize the multi-biomarker responses to single values, reflecting the integrated stress of PFOA on earthworms.

In the present study, we investigated the damages to the antioxidant defense system, lysosomal membrane stability and DNA in the earthworm *E. fetida* caused by exposure to PFOA in OECD artificial soils under standard laboratory conditions. The oxidative stress biomarkers analyzed in this study include SOD, CAT, glutathione peroxidase (GPx), GST, GSH and LPO. The aim of the present study was to systematically investigate and compare the multi-biomarker responses of *E. fetida* to PFOA in artificial soil and to provide valuable information for chemical risk assessment and early warning indicators of soils contaminated by PFOA.

## 2. Materials and methods

### 2.1 Soils and reagents

Artificial soil was prepared according to a standard method from OECD guideline no. 207.^27^ The composition of the OECD soil (dry weight) was a mixture of 70% quartz sand, 20% kaolinite clay, and 10% finely ground sphagnum peat, with pH adjusted to 6.5 by addition of calcium carbonate. The soil pH measurements were performed according to ISO 10390: 2021.^43^

Perfluorooctanoic acid (98% purity) was purchased from Alfa Aesar China Co. (Tianjin, China). The stock solution (1000 mg L^−1^) was prepared by dissolving PFOA in dimethyl sulfoxide (DMSO, 0.005%, v/v) and stored at 4 °C. Ultrapure water (18 MΩ) was obtained by using a Milli-Q water purification system (Millipore, USA). Chemicals used for biomarker analysis were obtained from Sigma-Aldrich China Co. (Shanghai, China). All other reagents were of analytical grade and purchased from Beijing Chemical Co. (Beijing, China).

### 2.2 Earthworms

Earthworms *E. fetida* were purchased from Jinan Qingshun earthworm breeding farm (Jinan, China) and were acclimated in clean artificial soils for 7 days under controlled conditions (22 °C, 12 h light/12 h dark cycle, 60–80% humidity) prior to the experiments. We selected healthy earthworms with well-developed clitellum, weighing 350–450 mg, and 3 months old for toxicity testing. The study protocol regarding earthworms was in accordance with national and institutional guidelines for the protection of human subjects and animal welfare.

### 2.3 Experimental design

Based on the results of acute toxicity tests of PFOA to earthworms^30,37^ and our pre-experiments, six different concentrations of 0, 10, 20, 40, 80 and 120 mg kg^−1^ soil (dry weight) were used in this study. The 80 mg kg^−1^ was considered as 1/10 of 14d-LC_50_ for PFOA (about 800 mg kg^−1^ for earthworm *E. fetida* in soil). The maximum value of PFOA treatment level was 1.5 times of the 1/10 of 14d-LC_50_ amount. The lowest tested concentration was set as 10 mg kg^−1^, which is a little higher than the 1/100 of 14d-LC_50_ for PFOA. An exposure test model in artificial soil was used to conduct the experiment, and PFOA was applied as the chemical that induced multi-biomarker responses in the earthworms. The artificial soils were spiked with stock solution of PFOA so as to obtain the different exposure concentrations. The soil moisture content was adjusted to 65% of the water holding capacity with ultrapure water.^44^ The PFOA-spiked soils were placed in a 1 L glass beaker, with each beaker containing 750 g of soil. Tests were conducted in triplicate for each exposure concentration. The soils of control group were prepared in the same way with no PFOA added.

Twenty healthy earthworms with uniform body lengths and weights were transferred to the experimental soils after rinsing with ultrapure water to remove adhering soils or particles. The beakers were then sealed with plastic films containing micropores to allow ventilation as well as to prevent the earthworms from escaping. The culture condition was maintained at 22 °C, 60–80% ambient humidity with a 12 h light/12 h dark cycle in an artificial climate incubator. Earthworms were removed from the soil after 3, 7, 14, 28 and 42 days of exposure, rinsed, and maintained in Petri dishes with wet filter paper for 24 h to purge their gut contents. An appropriate amount of diet (5 g per beaker) was added to the soil surface at the start of the experiment and was supplemented weekly. No earthworms died during the experimental period.

### 2.4 Antioxidant defense biomarkers assay

Two randomly selected earthworms of each replicate beaker for the analysis of six oxidative stress biomarkers and protein content were weighted and then cooled on ice to facilitate dissection process. The earthworm tissue samples were taken, washed using ultrapure water, and then pooled. For the preparation of tissue extract, the samples were homogenized (1 : 4, w/v) in chilled Tris–HCl buffer (20 mM, pH 7.8) using a glass homogenizer. Homogenates were then centrifuged at 10 000*g* at 4 °C for 20 min, and the supernatants were collected for the determination of total protein, enzymatic activity, GSH content and LPO level.

SOD activity was assayed by the method interpreted by McCord and Fridovich^45^ and expressed as U mg^−1^ of total protein concentration. CAT activity (U g^−1^ protein) was analyzed by utilizing the method described by Aebi^46^ and measuring the decrease in absorbance at 240 nm because of the hydrogen peroxide consumption. GPx activity was quantified by the method proposed by Hafeman *et al.*^47^ and expressed as nmoles of GSH used by every milligram of protein per minute. GST activity was quantified by the method developed by Habig *et al.*^48^ and expressed as nmol min^−1^ mg^−1^ protein. GSH content (μmol g^−1^ protein) was determined by the fluorimetric method suggested by Hissin and Hilf.^49^ LPO level was quantified in term of malondialdehyde (MDA) (nmol mg^−1^ protein) according to the method described by Buege and Aust.^50^ Protein contents were measured by the method developed by Bradford^51^ and consulting bovine serum albumin as a standard.

### 2.5 Neutral-red retention time (NRRT) assay

Lysosomal membrane stability was assessed using the NRRT assay as described by Weeks and Svendsen.^34^ The stock solution of neutral red was obtained by dissolving 20 mg of neutral red powder in 1 mL DMSO. Then, 10 μL of the stock solution and earthworm physiological Ringer solution (2.5 mL) were mixed to obtain an 80 μg mL^−1^ neutral red working solution, which was renewed every hour during the measuring process to avoid crystallization. A volume of 20 μL of coelomic fluid containing coelomocytes was collected by inserting a hypodermic needle directly into the coelomic cavity posterior to the clitellum of the earthworm, and mixed with an equal volume of Ringer solution. The mixture (20 μL) was placed onto a clean microscope slide for 30 s, and then an equal volume of the neutral red working solution was pipetted after the cells adhered to the slide surface. Timing began after a cover slip was placed on the top. Each slide was observed under a light microscope at 400 times magnification in 2 min intervals followed by returning to a humidity chamber when not being observed. The observation was stopped when the ratio of cells with fully stained cytosols was over 50% of the total number of cells. This time was recorded as the NRRT (min).

### 2.6 Comet assay

Earthworm coelomocytes that were used for comet assays were collected using a non-invasive method with slight modification.^38,52^ Briefly, individual earthworms were rinsed in 1 mL of extraction medium composed of 5% ethanol, 95% saline, 2.5 mg mL^−1^ EDTA and 10 mg mL^−1^ guaiacol glyceryl ether (pH 7.3) for 3 min. After the extraction, the extrusion fluid was mixed with phosphate-buffered saline solution (PBS, 0.1 mol L^−1^, pH 7.4). The extruded coelomocytes were collected by centrifugation at 3000*g* at 4 °C for 10 min. PBS was then used to wash the cells prior to the comet assay.

The single-cell gel electrophoresis (SCGE), also known as comet assay, was performed as described originally by Singh *et al.*^53^ with slight modifications. A volume of 20 μL of coelomocytes suspension was mixed quickly with 80 μL of 0.7% low melting agar (LMA) in PBS at 37 °C and pipette onto fully frosted slides precoated with a layer of 80 μL 1.0% normal melting agarose (NMA). After solidification, the slides were placed in fresh lysis buffer (2.5 M NaCl, 100 mM Na_2_EDTA (pH 10.0), 10 mM Tris–HCl, 1% sodium *N*-lauroylsarcosinate, 1% Triton X-100 and 10% DMSO) for 1 h at 4 °C, and then flushed by ultrapure water. The slides were then embedded in an electrophoresis tank with freshly made alkaline buffer (300 mM NaOH, 1 mM Na_2_EDTA) for 20 min to unwind the DNA. Electrophoresis was then performed for 25 min by applying an electric field of 25 V (1 V cm^−1^) and adjusting the current to 300 mA. The slides were then neutralized (0.4 M Tris–HCl, pH 7.5) thrice at 5 min intervals and stained with ethidium bromide (2 mg mL^−1^) for fluorescence microscopy analysis. CASP software was used to obtain various parameters including tail DNA%, tail DNA length (TL), and olive tail moment (OTM).

### 2.7 IBR calculation and statistical analysis

IBR was applied in this study to combine multi-biomarker responses in the earthworms (SOD, CAT, GPx, GST, GSH, LPO, LMS and OTM) into an index according to Beliaeff and Burgeot,^42^ which is accepted as a measurement of integrated stress of toxicants.^54^ In this study, the IBR value indicated the toxically induced stress of PFOA on earthworms. OTM was considered as representative biomarker to quantify the extent of DNA damage and was used in the IBR calculation. The IBR index was calculated by summing up triangular star plot areas calculated for each two neighboring biomarkers in the dataset. The basis of data processing of each biomarker was described as follows: (1) the mean and standard deviation (SD) for each sample was calculated; (2) data was standardized *via* the equation *Y*_i_ = (*X*_i_ − *m*_i_)/*S*_i_, where *Y*_i_ is the standardized value of a biomarker, *X*_i_ refers to the mean value of a biomarker for each sample, *m*_i_ and *S*_i_ represent the mean value and SD of a biomarker calculated for all samples, respectively; (3) *Z*_i_ value was computed *via* the equation *Z*_i_ = *Y*_i_ or *Z*_i_ = −*Y*_i_ on the condition that the biomarker was induced or inhibited, and then the score (*B*_i_) for a given sample was computed as *B*_i_ = *Z*_i_ + |*Z*_min_|, where |*Z*_min_| is the absolute value of the minimum value in the dataset. In the present study, eight scores for each sample (*B*_1_–*B*_8_) were expressed in the form of star plots, and the corresponding IBR value was calculated as the area of the plots. Because the biomarker arrangements on the star plots generated different IBR values,^40^ all the sequences of the eight biomarkers were taken into account in this study and the average value of all types of IBR values was calculated as the final result.

All statistical analysis in this study was performed using the SPSS software (version 20.0, SPSS Inc.), and the results were expressed in the form of mean ± SD. One-way analysis of variance (ANOVA) followed by Tukey's post hoc test were conducted to evaluate the significance of differences between control and specific treatments at the *P* < 0.05 level. The software Origin 9.0 was used for graphical rendering.

## 3. Results

### 3.1 Weight change

**Fig. 1:**
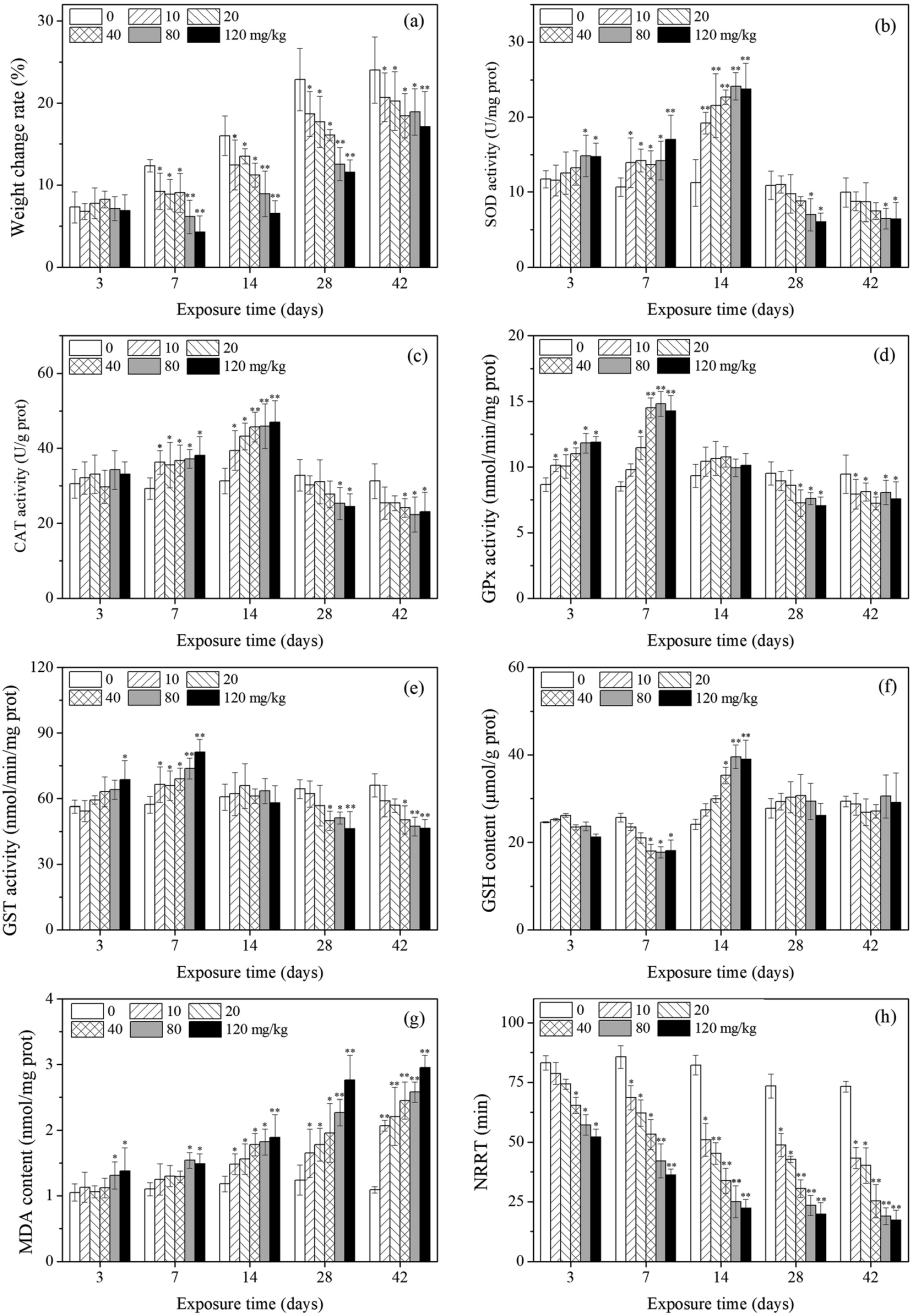
Effects of PFOA on weight change rate (a), oxidative stress biomarker responses (b)–(g), and NRRT (h) in *E. fetida* following 42 days exposure. Data are expressed as mean ± SD (*n* = 3). Significant difference *versus* control group: *P < 0.05, **P < 0.01. Two earthworms were used for biomarker assay in each triplicate at each exposure time point.

The effects of PFOA on earthworm weight are presented in Fig. 1a. The weight change rate of the earthworms in the exposure groups were significantly lower compared with control groups during the experimental period except for earthworms exposed to PFOA for 3 d. After exposure for 7 d, the growth of the earthworms of all treatment groups were all significantly inhibited in comparison with the control groups.

### 3.2 Antioxidant responses-enzymatic system

As shown in Fig. 1b, SOD activity changed with PFOA concentration and exposure time, showing a trend of activation at the beginning and an inhibition towards the end. Compared with the control, SOD activity was significantly induced after 3 days of exposure to PFOA in high concentration groups (80 and 120 mg kg^−1^). By day 7 and 14, the SOD activity of all treatments was significantly higher or extremely higher relative to the control. During the subsequent exposure times (days 28 and 42), SOD activity was inhibited and significant decrease was observed in the high PFOA treatments (80 and 120 mg kg^−1^).

Changes in CAT activity are presented in Fig. 1c. The CAT activity of earthworms showed a trend which was similar to that of SOD activity, despite no significant change was observed after 3 days of exposure. In comparison with the control, CAT activity of earthworms significantly increased from 7 to 14 days. After 28 days of exposure, CAT activity decreased with increasing PFOA concentration.

The GPx activity are shown in Fig. 1d. A significant increase in GPx activity was observed after 3 and 7 days of PFOA exposure when compared to the control, except in the 10 mg kg^−1^ group by day 7. The enzyme activity was not markedly different relative to the control on day 14. After 28 days of exposure, GPx activity in earthworms significantly decreased, except in the low treatment groups (10 and 20 mg kg^−1^) on day 28.

The variation trends of GST activity in the different PFOA doses are shown in Fig. 1e. After 3 days of exposure, GST activity was induced only in the highest concentration group (120 mg kg^−1^). However, all the exposure group exhibited significant increase on day 7 compared with control. After 28 days of exposure, GST activity was inhibited by increasing PFOA concentration in soils, with the significant inhibition rates in the presence of 40, 80 and 120 mg kg^−1^ PFOA.

### 3.3 Antioxidant responses-nonenzymatic system

Changes in the GSH content in *E. fetida* are shown in Fig. 1f. In general, the GSH content in the treatment groups decreased first and then intensively increased as the exposure time prolonged, and then decreased to the control level at the later stage. The significant increased GSH content appeared on day 14 in high concentration groups (40, 80 and 120 mg kg^−1^), while the significant decreased value exhibited on day 7 also in the high treatments.

The effect of different PFOA concentrations on the LPO level in earthworms are shown in Fig. 1g. A trend of increase throughout the entire experimental period was observed, except in the low treatments (10, 20 and 40 mg kg^−1^) during early exposure (3 and 7 d). From 14 to 42 days of exposure, LPO level was stimulated by increasing PFOA concentrations and experimental duration.

### 3.4 Lysosomal membrane stability

As shown in Fig. 1h, the NRRT decreased with increasing concentration during the entire exposure period, and statistically significant differences were found for all treatments except for the 10 and 20 mg kg^−1^ PFOA treatments on day 3. In addition, the NRRT also decreased with the prolong of exposure periods. At the early stage of exposure (3 days), 40 mg kg^−1^ PFOA induced damage to the lysosomal membrane, decreased the NRRT from 83.2 min (control) to 65.3 min, and the NRRT was even lower at higher PFOA exposure groups. In the late experiment stage (42 days), the NRRT for all PFOA treatments decreased from 73.2 min (control) to 43.3, 40.2, 25.3, 18.9, and 17.2 min at the doses of 10, 20, 40, 80 and 120 mg kg^−1^, respectively, and the NRRT value obtained in the highest treatment was 4.26 times shorter than that of the control.

### 3.5 DNA damage

**Fig. 2:**
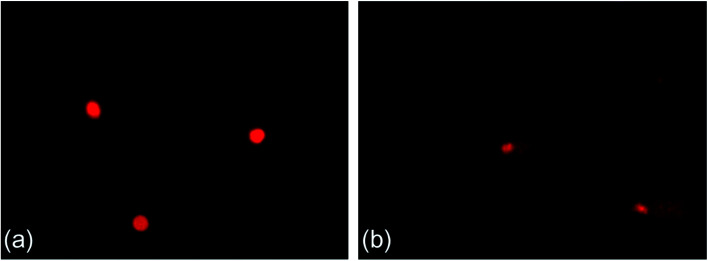
Typical comet figures. (a) Control and (b) 120 mg kg^−1^ PFOA on day 42. Two earthworms were used for comet assay in each triplicate at each exposure time point.

**Fig. 3:**
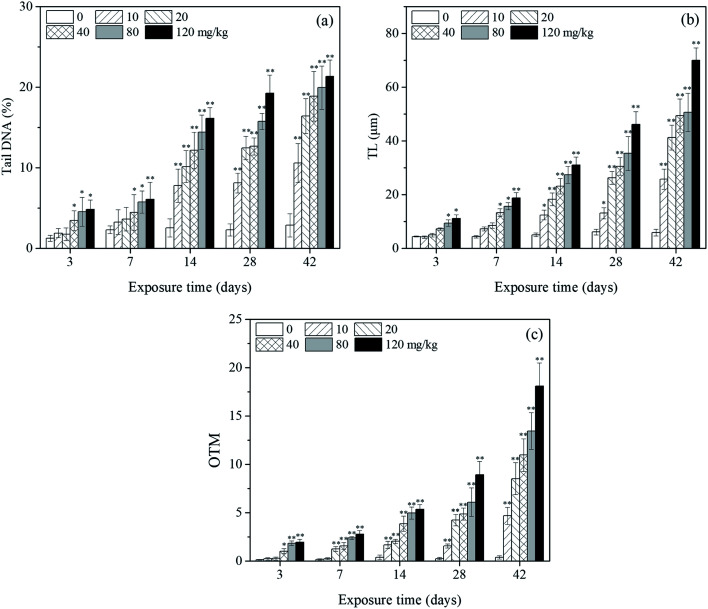
Effects of PFOA on tail DNA% (a), TL (b) and OTM (c) values in *E. fetida* following 42 days exposure. Data are expressed as mean ± SD (*n* = 3). Significant difference *versus* control group: *P < 0.05, **P < 0.01. Two earthworms were used for comet assay in each triplicate at each exposure time point.

Fig. 2a shows a typical comet image of undamaged DNA from earthworm coelomocytes, and Fig. 2b shows a typical comet image of damaged DNA of earthworm coelomocytes after exposure to PFOA in soils. The comet head centers were rounded, dense, and shiny in the control group. As the exposure concentration increased, the migration and expansion of DNA in the nucleus became more obvious. The dynamic changes of tail DNA%, TL and OTM in *E. fetida* exposed to PFOA are shown in Fig. 3. The change in OTM value was in consonance with that of tail DNA% and TL. All exposure groups increased significantly with PFOA treatment during the entire exposure period except for 10 and 20 mg kg^−1^ PFOA on day 3, and 10 mg kg^−1^ PFOA on day 7. As shown in the figure, the increases exhibited a concentration-related and exposure time-related effect. The DNA damage was more pronounced after 14 days of exposure, and tail DNA%, TL and OTM reached a maximum value after 120 mg kg^−1^ PFOA exposure on day 42, which was 7.44, 11.81 and 47.66 times higher than that of the control, respectively.

### 3.6 IBR index

**Fig. 4:**
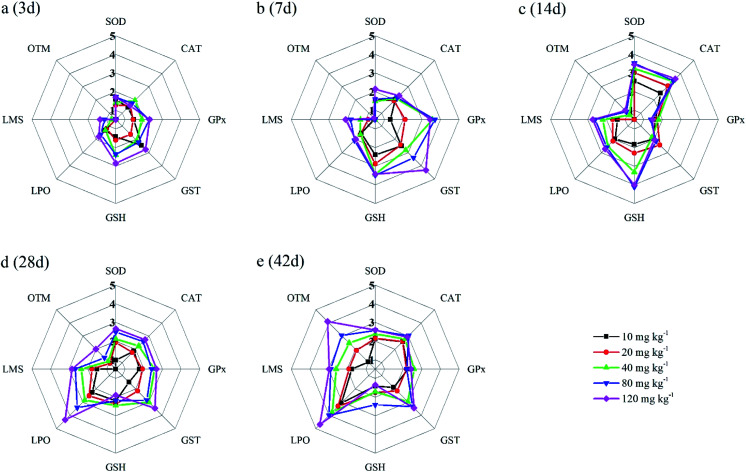
The star plots for the IBR calculation for the earthworm *E. fetida* exposed to PFOA: 3 days (a); 7 days (b); 14 days (c); 28 days (d); 42 days (e). Two earthworms were used to collect data for the star plots and IBR calculation in each triplicate at each exposure time point.

**Fig. 5:**
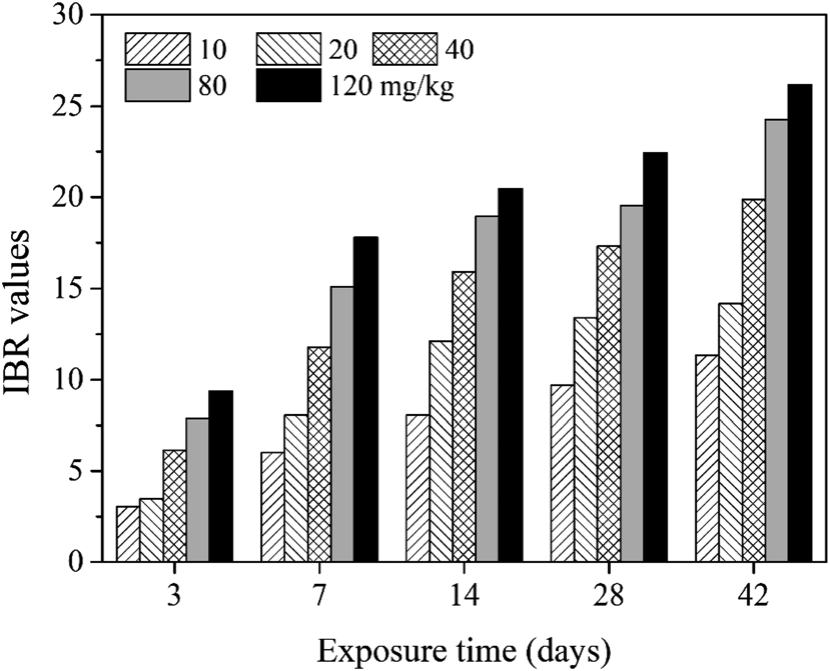
The IBR index calculated in *E. fetida* following 42 days exposure in PFOA contaminated soils. Two earthworms were used to collect data for IBR calculation in each triplicate at each exposure time point.

In the present study, eight biomarkers were chosen to calculate IBR index in order to explore if sub-chronic toxicity occurs in earthworms exposed to PFOA. The star plots for the calculation of IBR index in the earthworm exposed to varying concentrations PFOA are shown in Fig. 4, and the obtained values of IBR are shown in Fig. 5. During the entire exposure period, an obvious increase of IBR values was observed except for 10 mg kg^−1^ PFOA by day 3. In addition, a concentration-related and exposure time-related effect can be observed in the IBR calculation results.

## 4. Discussion

Earthworm biomarkers have been widely investigated to explore the effects of organic pollutants on the organisms in the past few years.^26,55^ The main indicators used in many studies were survival, growth, reproduction, avoidance behavior, *etc.* These biomarkers are of physiological and individual level and usually respond to toxic substances only at high levels. Bodyweight is one of the macroscopic indicators that shows the effect of pollutants on organisms although it usually exhibits an insensitive response in toxicological tests.^56^ In the present study, earthworm weight in the exposure group was significantly reduced compared with control after exposure for 7 d, indicating that the PFOA concentrations adopted in this study significantly affected the earthworms' growth, which may be related to changes of biomarker responses at molecular, subcellular and cellular levels. In another study conducted by Zheng *et al.*,^37^ growth inhibition of earthworm *E. fetida* was detected after 14 d acute exposure of 50–800 mg kg^−1^ PFOA in OECD soils, and significant increase of growth inhibition rates were observed, showing a dose–response relationship.

In comparison with the above macroscopic biomarkers, the biochemical responses at the molecular or subcellular level are sensitive to response even under the effect of a low concentration of pollutants and are closely related to the toxicology mechanisms of pollutants, which makes them more suitable for toxicology research.^57^ In the present study, 6 kinds of oxidative stress biomarkers were examined as molecular biomarkers, while LMS and DNA damage in earthworm coelomocytes were used as biochemical indicators at subcellular level. These biomarkers have been proved to be sensitive, reliable and dose-related in previous studies using earthworm as bioindicators.^36,40,58^ However, few cases have focused on the multi-biomarker responses at different levels simultaneously, and the investigation of integrated biomarker responses in earthworm exposed to PFOA has not been reported.

Reactive oxygen species (ROS) which comprise a series of free oxygen radicals are short-lived chemical species containing unpaired electrons, formed by the partial reduction of molecular oxygen.^59^ These substances are highly reactive and can attack various kinds of biomolecules in their vicinity, which is known as “oxidative stress”.^60^ Normally, the generation and elimination of ROS in organisms are in a dynamic equilibrium. However, the balance could be disturbed due to the overproduction of ROS or the deterioration of the antioxidant system.^61^ Many previous studies have found that exogenous pollutants could induce oxidative stress in earthworms by the generation of excessive ROS, which causes a series of adverse effects on organisms including lipid, carbohydrate, protein and DNA damage.^62^ In order to counteract oxidative damage, an intricate antioxidant defense system is evolved in organisms involving ROS-scavenging enzymatic and nonenzymatic components. The enzymatic system includes SOD, CAT, GPx, GST and other antioxidant enzymes, while nonenzymatic system comprises GSH, oxidized glutathione (GSSG) and other nonenzymatic antioxidants.^63^ The typical antioxidant biomarkers used in the present study were often employed in biomonitoring programs to indicate ROS production.^64^

SOD is an important antioxidant enzyme that catalyzes the decomposion of superoxide radicals to H_2_O_2_ and oxygen. However, hydrogen peroxide is still toxic to cells and can be subsequently detoxified by CAT to water and oxygen.^65^ Therefore, SOD and CAT act as the first antioxidative defense line in scavenging superoxide radicals.^66^ In this study, similar trend was found for SOD and CAT activity, namely, both activated in the first two weeks of exposure and inhibited during the subsequent exposure period. In the early stage of the experiment, the activities of both SOD and CAT in earthworms increased with PFOA dose and exposure time, indicating that the accumulated PFOA in organisms gradually induced the generation of superoxide radicals. Therefore, the SOD activity needed to be enhanced to catalyze the superoxide radicals into H_2_O_2_, which subsequently led to the induction of the CAT activity, indicating a protective strategy against slight oxidative stress. However, under long-lasting contamination conditions, excess toxicity of accumulated oxidizing agents resulted in enzyme inhibition, exhibiting oxidative damages in organisms. A similar changing trend of SOD and CAT activities in earthworms has also been observed in a study conducted by Zhao *et al.*,^31^ that is, initially increased and then deactivated during a 28 days exposure of 5–40 mg kg^−1^ PFOA contaminated soils.

The contamination stress also leads to the generation of organic hydroperoxides (ROOH), another representative ROS, which could be decomposed by GPx and GST, consuming GSH and generate oxidized glutathione (GSSG) simultaneously.^67^ In this study, GPx and GST showed a similar response pattern, indicating that these enzymes operate together in the process of scavenging ROOH. During the early stage of the experiment, GPx and GST in earthworms were both stimulated, indicating that PFOA led to the generation of ROOH after entering into the earthworms and therefore induced detoxification reactions catalyzed by the two enzymes. But we have also noticed that, GPx activity of earthworm showed more obvious increase than that of GST during the early exposure period, especially by the 7th day of exposure period. Considering that another detoxification function of GPx is to catalytically degrade H_2_O_2_ to water and O_2_, such induction exhibited the effect of GPx on eliminating hydrogen peroxide generated with the accumulation of PFOA in the earthworms. As the exposure time prolonged, the activities of GPx and GST decreased until they were inhibited relative to control, suggesting that the damages on the organisms exceeded the scavenging capacity of the antioxidant defense enzymes. Prolonged or high doses exposure to contaminated soil could adversely affect organisms and cause a decrease in enzymatic activity.^68^

GSH is a tripeptide that contains an unusual peptide linkage between the carboxyl group of the glutamate side chain and the amine group of cysteine. It exists widely *in vivo* as an important detoxification substance, and plays a crucial role in coordinating the antioxidant defense processes.^38^ Many previous studies have proved that GSH could reduce PFOA oxidative stress in organisms.^69,70^ PFOA causes oxidative stress *via* ROS generation, which is reduced by the action of antioxidant enzymes with the consumption of GSH. As a result of the biochemical reaction, GSH is decreased and oxidized to GSSG. In the present study, the content of GSH in the earthworms in high exposed groups decreased significantly on day 7, implying the over-consumption of GSH in order to overcome the prevailing oxidative stress. When excessive GSH were consumed, the organisms would enhance the synthesis of GSH as the adaptation to the oxidative stress. Therefore, the content of GSH in the earthworms of high PFOA treatments rose markedly on day 14, suggesting a contaminant-induced adaptive response. At the later stage of the experiment, the GSH content of all exposure groups decreased to the control level, which may be related to the inhibition of the oxidative enzymatic activities under long-term contaminant stress.

LPO results from ROS oxidation under oxidative stress, generating a wide variety of metabolites including MDA, lipid hydroperoxides, hexanal, propanal, 4-hydroxy-2-nonenal, *etc.*^60,71^ These lipid peroxidation products may cause a variety of cell damage.^72^ Therefore, LPO has been widely used as a biomarker for oxidative damage. MDA is one of the ultimate lipid peroxidation metabolites in the cells, and thus, the LPO level can be indirectly measured *via* MDA.^60^ In this study, the MDA content rose significantly throughout the exposure duration in high level treatments, indicating that the PFOA accumulated in the organisms induced oxidative toxicity which exceeded the antioxidant defense capacity of the earthworms and caused oxidative damages. At the later stage of the experiment, extremely higher LPO level was observed in the five treatment groups, suggesting that all of the concentrations of PFOA caused high oxidative toxicity and resulted in a decreased earthworm growth rate and impaired lysosomal membrane, DNA and physiological functions. Similar results were observed in a previous research conducted by Xu *et al.*,^38^ who found that the MDA content increased in earthworms exposed to PFOS in soils, and the inductive effects were significant with increasing dose and extension of exposure time.

Lipid peroxidation could induce cell damage by generating peroxides, increasing the permeability of membranes and destroying the membrane structure. Therefore, lysosomal membrane stability is regarded as a useful biomarker of subcellular level for the action of toxicants, and NRRT assay has been proved to be reliable, dose-related, and practical in assessing the adverse effects of environmental pollution by using various earthworm species.^73–75^ The reduced NRRT value suggested that exposure stress has caused actual damages on lysosomal membrane of the organisms.^34^ In this study, the NRRT values decreased with increasing PFOA concentration during the entire exposure period, suggesting that the retention time of lysosomal staining could indicate the toxic effects of PFOA on earthworms. With the increase of PFOA concentration in soils and the extension of exposure time, the effect on the lysosomal membrane in the organisms was more obvious. Svendsen *et al.*^75^ also proved that the LMS in earthworm coelomocytes was sensitive to various pollutants, and a potential mechanism of the toxicity was due to the dysfunction of lysosomal membranes in organisms. The NRRT can indicate the adverse effects of exogenous contaminants on earthworms at sublethal doses by detecting the change in lysosomal membrane vulnerability in earthworm coelomocytes after exposure to toxic substances.^76^

The comet assay has been used to asses DNA damage in organisms caused by exposure to perfluoroalkyl substances in environmental monitoring and research programs.^77,78^ Zheng *et al.*^37^ used an artificial soil method to study the effects of PFOS and PFOA on earthworms, and DNA damage was detected in the organism after exposing to the two perfluoroalkyl substances. Xu *et al.*^38^ found that PFOS could damage earthworm coelomocytes by using the filter paper method; the OTM, tail DNA% and TL values increased significantly with the PFOS concentration. ROS would be generated when earthworms were stressed by contaminated soils spiked with PFOA, and the ROS can cause oxidative damage, which leads to DNA strand breakages and chromosome aberration.^37^ In the present study, tail DNA%, TL and OTM values of the treatment groups were significantly elevated relative to the control except for low treatments during the early stage of the experiment, indicating the occurrence of DNA damage and genotoxicity of PFOA to the earthworms. Moreover, a positive dose–response relationship was observed among PFOA concentration, exposure time and the corresponding tail DNA%, TL and OTM values. In consideration of the increased LPO and decreased LMS in the earthworms with the prolonged exposure time, we speculate that the enhanced DNA damage observed in earthworms exposed to PFOA was due to oxidative stress, implying that ROS accumulation in the organisms caused subsequent DNA damage.

In this study, the responses of different biomarkers in earthworms to PFOA exposure are inconsistent. Therefore, the use of one bioindicator alone may be not effective enough for the comprehensive evaluation of the integrated toxic effect of PFOA. IBR index is a qualitative tool for assessing toxic effects and pollution levels under different exposure conditions by the combination of multi-biomarker responses into a star plot and numeric value.^42^ Although the IBR cannot be used for the quantitative evaluation of pollution stress degree in model organisms, the calculation procedure of IBR is not limited to the type and number of integrated biomarkers, which makes it possible to be widely applied in many field and laboratory studies to evaluate environmental risk.^79–81^ In addition, the star plots for IBR calculation can be used as a useful graphic aid for exploratory analysis of data in a multi-biomarker approach. In our previous study,^40^ the IBR index was applied to compare the toxicity of different arsenic species by using earthworm *E. fetida* in an artificial soil test, and the toxicity of the four arsenic species was obviously distinguished. In this study, among the three parameters used to quantify DNA damage, the OTM corresponding to the distance between the center of a comet head and tail was considered to be particularly sensitive to quantify the extent of DNA damage,^82^ and was therefore selected in the IBR calculation. In the present study, the earthworms exposed to PFOA suffered increased levels of stress during the entire exposure period, exhibiting a concentration-related and exposure time-related effect. The results are in accordance with those concluded by Zhao *et al.*,^81^ who also observed a good agreement between IBR values and organic pollutant levels. Furthermore, the scores of the eight biomarkers exhibited in the star plots showed a different changing trend. At the early stage of the experiment, as shown in Fig. 4a and b, biomarkers of antioxidant defense systems exhibited higher scores in the star plots. However, as the exposure time prolonged, the scores of the bioindicators which reflect the damage to the earthworms, including LPO, LMS and OTM, increased markedly and led to elevated IBR values, as exhibited in Fig. 4d and e. The results proved that PFOA is toxic to earthworms under either short or long-term exposure condition even at the lowest exposure level. Moreover, according to graphical changing trend of the IBR star plots, along with the multi-biomarker responses, it can be speculated that the biomarkers of antioxidant defense system are sufficiently sensitive for short-term PFOA biomonitoring programs, while the bioindicators that indicate actual damage in organisms are more suitable to be employed in long-term monitoring programs for the risk assessment of PFOA.

In the present study, the multi-biomarker responses in the earthworms were not measured by using gene expression analysis techniques. Therefore, there is no direct evidence to tie PFOA to a particular enzyme or transcription factor, which is the limitation of such toxicology proposals. The technique proposed in this study is actually non-targeted for assessing general toxicity of PFOA in soil. In order to investigate earthworm genes that are turned on or off by PFOA, the mRNA isolation, sequencing, transcriptome assembly followed by differential gene expression studies should be conducted in the next stage. The plan for future research is to validate highly specific genes in earthworm that are altered by PFOA, which can be used as sensitive biomarkers to detect sub-lethal concentrations of PFOA in the soil.

## 5. Conclusions

In this study, we systematically investigated the damages to the antioxidant defense system, lysosomal membrane stability and DNA in the earthworm *E. fetida* caused by exposure to PFOA in artificial soils under standard laboratory conditions. The results of multi-biomarker responses indicated that PFOA can induce various adverse effects on earthworms, including growth inhibition, oxidative stress and genotoxicity, resulting in lipid membrane peroxidation, decreased lysosomal membrane stability and DNA damage. LPO, LMS and DNA damage all presented dose- and time-dependent relationships. The IBR index showed that the integrated stress induced by PFOA increased markedly throughout the exposure period, exhibiting a concentration-related and exposure time-related effect. The graphical changing trend of the IBR star plots, along with the multi-biomarker responses, suggested that the biomarkers of antioxidant defense system in earthworms are sufficiently sensitive for short-term PFOA biomonitoring programs, while the bioindicators that indicate actual damage in organisms are more suitable to be employed in long-term monitoring programs for the risk assessment of PFOA. Our results showed that PFOA can potentially damage soil ecosystems, which provides valuable information for chemical risk assessment of PFOA in the soil environment and early warning bioindicators of soils contaminated by PFOA.

## Conflicts of interest

The authors declare that there are no conflicts of interest.

## Supplementary Material
